# 3D Printable Electrically Conductive Hydrogel Scaffolds for Biomedical Applications: A Review

**DOI:** 10.3390/polym13030474

**Published:** 2021-02-02

**Authors:** Sandya Shiranthi Athukorala, Tuan Sang Tran, Rajkamal Balu, Vi Khanh Truong, James Chapman, Naba Kumar Dutta, Namita Roy Choudhury

**Affiliations:** 1 School of Engineering, RMIT University, Melbourne, VIC 3000, Australia; S3801296@student.rmit.edu.au (S.S.A.); s3708733@student.rmit.edu.au (T.S.T.); rajkamal.balu@rmit.edu.au (R.B.); 2 School of Science, RMIT University, Melbourne, VIC 3000, Australia; vi.khanh.truong@rmit.edu.au (V.K.T.); james.chapman@rmit.edu.au (J.C.)

**Keywords:** 3D printing, hydrogels, conductive polymers, graphene, tissue engineering, bioelectronics

## Abstract

Electrically conductive hydrogels (ECHs), an emerging class of biomaterials, have garnered tremendous attention due to their potential for a wide variety of biomedical applications, from tissue-engineered scaffolds to smart bioelectronics. Along with the development of new hydrogel systems, 3D printing of such ECHs is one of the most advanced approaches towards rapid fabrication of future biomedical implants and devices with versatile designs and tuneable functionalities. In this review, an overview of the state-of-the-art 3D printed ECHs comprising conductive polymers (polythiophene, polyaniline and polypyrrole) and/or conductive fillers (graphene, MXenes and liquid metals) is provided, with an insight into mechanisms of electrical conductivity and design considerations for tuneable physiochemical properties and biocompatibility. Recent advances in the formulation of 3D printable bioinks and their practical applications are discussed; current challenges and limitations of 3D printing of ECHs are identified; new 3D printing-based hybrid methods for selective deposition and fabrication of controlled nanostructures are highlighted; and finally, future directions are proposed.

## 1. Introduction

**Figure 1:**
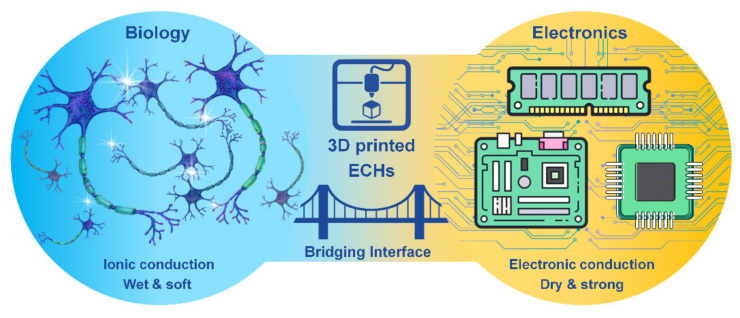
3D printed electrically conductive hydrogels (ECHs) as the bridging interface of biology and electronics.

Hydrogels are a three-dimensional (3D) network of crosslinked hydrophilic polymers with high water sorption properties. Electrically conductive scaffolds (ECSs), including electrically conductive hydrogels (ECHs) are potential candidates for biomedical applications, such as bioelectronics, drug delivery and tissue engineering of skin, muscle, cardiac, nerve and bone tissues [1]. ECHs have been at the frontline of “smart conductive biomaterials” development due to their resemblance to biological tissues in terms of mechanical properties, water retention, bioactivity and other extracellular matrix-like properties [2]. ECHs have made it possible to minimize the property mismatch at bioelectronic interfaces, providing wet and ion-rich physiological environments in a 3D nanostructured conductive network, offering an extremely high surface area for seamless bio-integration that is difficult to accomplish on a conventional electronic interface [3]. Moreover, ECHs as electrodes can promote signal transductions between biological and electrical circuits by accurately controlling/allowing localized amplification and/or filtering of bio-derived signals [4], and an improved cell adhesion, proliferation and differentiation can be achieved with electrical stimulation [5]. However, achieving desired conductivity and mechanical properties (e.g., toughness and stretchability) is the key obstacle in formulating ECHs for bioelectronics while retaining biocompatibility. In addition, the integration of other features, such as wet adhesion along with self-healing and shape memory features, into ECHs is also crucial to many functional applications of hydrogel bioelectronics and implantable devices [6]. Although metallic electrodes such as gold, platinum, glassy carbon etc. are used as implantable devices, their usage often leads to poor long-term stimulation and recording performances. Thus, significant challenges exist for developing conducting polymer gels for neural interfaces, which require intimate contact between the excitable tissue and the electrode for stimulation of cells, which is often limited by the presence of extracellular fluid through which the signal transmission occurs. ECH offers the potential to support the intimate contact between the tissue and the electrode and combine the best of both worlds of biology and electronics (Figure 1).

A wide variety of ECHs have been synthesised to date by mixing various types of conventional insulating polymer matrices (providing structural support and water sorption properties) with conductive polymers or filler materials (providing electrical conductivity) [5]. Among electrically conductive polymers, poly(3,4-ethylenedioxythiophene) (PEDOT), polyaniline (PANI) and polypyrrole (PPy) are attractive materials for biomedical applications due to their biocompatibility (including cell viability, adhesion, proliferation and differentiation), and tuneable electrical conductivity (by doping). However, their tissue engineering applications are often limited by their poor processability and mechanical brittleness, which has led to the development of several conductive polymer-based hybrid ECHs [7]. On the other hand, electrically conductive fillers, such as carbon-based materials, transition metal carbides/nitrides and liquid metals provide highly efficient electron transport channels across polymer matrix (having covalent/or non-covalent interactions with polymer chains) to achieve high conductivity [8]. Over the last decade, graphene-based materials have drawn exceptional attention as conductive fillers for ECH composites due to their natural abundance, outstanding electrical conductivity, mechanical properties, and cell adhesion, proliferation and differentiation support qualities [9]. In recent years, MXenes and liquid metals have gained increasing research interest in the field of biomedical engineering due to their unique combination of properties, including hydrophilicity or high fluidity, metallic conductivity and good biocompatibility [10,11]. Such conductive filler materials not only offer the flexibility of tuning desired structure and physicochemical properties of ECHs but also influence rheological properties of inks during 3D scaffold fabrication.

**Figure 2:**
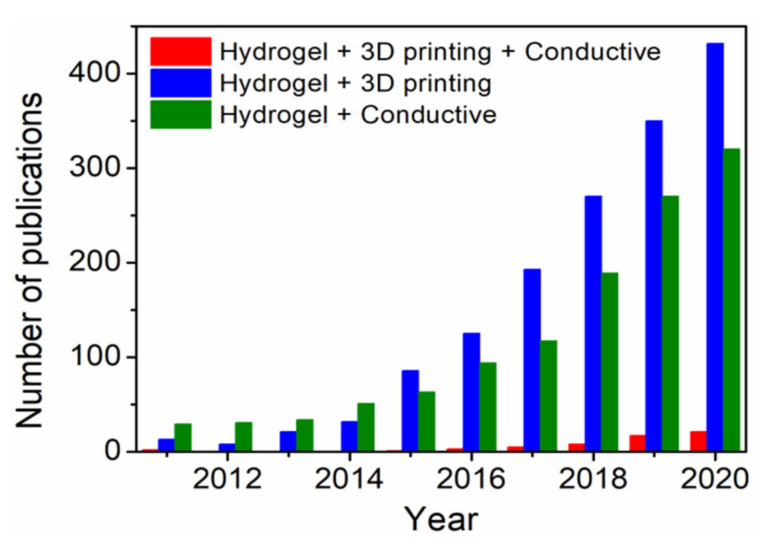
Publication trends over the last ten years obtained with keywords “Hydrogel”, “3D printing”, “Conductive” and their combination using Web of Science.

Several methods for fabrication of 3D scaffolds, such as solvent casting, moulding, electrospinning and 3D printing have been reported in the literature [12]. In particular, 3D printing of hydrogels has gained increasing research attention in recent years as rational design strategy for emerging biomedical applications, where the technology involves layer-by-layer fabrication of digitally derived 3D model objects through progressive addition of materials as inks [13]. Moreover, hydrogel-based 3D bioprinting offers the advantage of constructing living structures from bioinks containing live cells, growth factors and other biocompatible materials [14]. In combination with 3D bioprinting, fabrication of 3D ECHs might be one of the most advanced approaches towards next-level bioelectronics regarding potential functionalities and design possibilities. However, the scientific community still faces numerous challenges in synthesis, 3D printing and crosslinking of electrically conductive materials (inks). For more than a decade, ECHs themselves have been studied for potential tissue engineering and biosensor applications [5], while the 3D printing of such functional materials has been investigated only recently [15]. Moreover, in the past five years, there has been a significant rise in the number of publications (Figure 2) on “3D printing hydrogels” and “conductive hydrogels”. However, the number of publications on 3D printing of conductive hydrogels has been much lower than the overall number of publications in the general field of “3D printing hydrogels”. In this review, recent achievements, challenges and future perspective in 3D printing of ECHs comprising electrically conductive biocompatible polymers (PEDOT, PANI and PPy) or fillers (graphene, MXenes and liquid metals) that have potential applications in the field of biomedicine, including tissue engineering, bioelectronics and medical devices, are summarized.

## 2. Mechanism of Electrical Conductivity in ECHs

**Figure 3:**
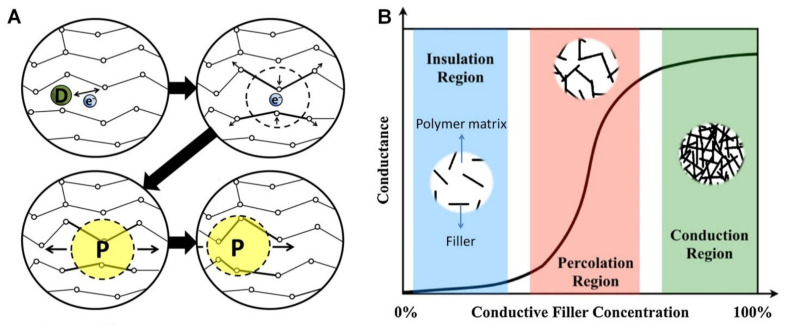
(**A**) Schematic of electrical conductivity of conductive polymers; where, the dopant D creates a delocalized charge (by adding or removing an electron to/from the polymer chain), which is then localized (energetically favourable) and surrounded by a local distortion of the crystal lattice, creating polaron P (a radical ion associated with a lattice distortion), which then travels along the polymer chain to conduct electricity. Adapted with permission from Ref. [16]. Copyright 2014, Elsevier. (**B**) Schematic of percolation behaviour and electrical conductance as a function of conductive filler concentration in an insulting matrix. Adapted with permission from Ref. [3]. Copyright 2019, Elsevier.

Electrical conductivity (or its inverse, electrical resistivity), is a fundamental material property that quantifies how strongly it conducts or resists electric current. The overall electrical conductivity of ECHs swollen in biological fluids (electrolytes) comes from the electronic functionality of conductive polymers and the ionic contribution and depends on the inter- and intra-chain charge transfer along polymer chains throughout the matrix/networks [16]. The electronic conductivity of ECHs relies on the conjugated backbone, formed by a series of π-bonds and strongly localized σ-bonds, of the conductive polymer (e.g., PEDOT, PANI and PPy) chains. During polymerisation, the p-orbitals in the series of π-bonds overlap each other, triggering electron redistribution. The delocalized electrons are allowed to move freely within the polymeric backbone, inducing intrinsically electronic conductance into the conductive polymers [16]. Similar to the ionic conductivity, the electronic conductivity is often improved by ionically conductive dopants. Their purpose is to introduce a charge carrier into the conductive polymer networks, disrupting the stable crystal lattice backbone and allowing charge to travel along the polymer chain in the form of polarons or bipolarons [17]. For instance, while PANI has a low conductivity of ~10^−9^ S cm^−1^ in its emaraldine form, its protonated salts with polaron and bipolaron states achieve much higher conductivity of ~10^−1^ S cm^−1^ [18]. A schematic of electrical conductivity of conductive polymers is shown in Figure 3A. In ECHs comprising insulating polymer matrix with 3D interpenetrating networks of fillers (e.g., graphene and MXenes) with inherent electrical conductivity, the mechanism of electron transfer can be described by percolation theory [3]. The percolation behaviour and change in electronic conductance of the system as a function of conductive filler concentration can be conceptualized as shown in Figure 3B. In the insulation region, almost no conduction occurs due to insufficient volume fraction of fillers to create an effective electron transfer pathway. whereas when percolation threshold is reached with the increase in the concentration of fillers, conductivity increases (several orders in magnitude) and finally reaches stable conduction region with further increase in concentration of fillers forming a stable interpenetrating network structure [3].

## 3. 3D Printing of ECHs

**Figure 4:**
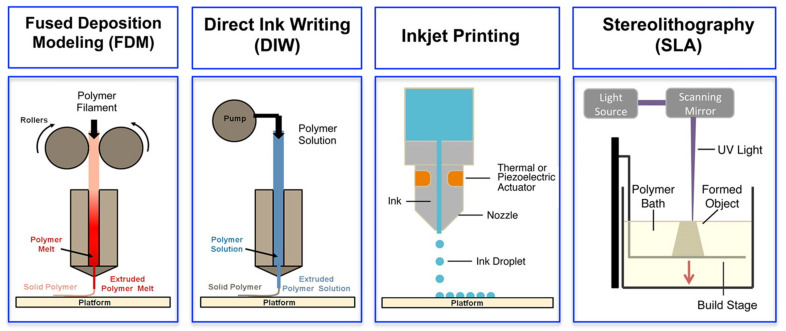
Schematics of different 3D printing methods: extrusion-based methods, such as fused deposition modeling (FDM) and direct ink writing (DIW), inkjet printing and light-based stereolithography (SLA). Adapted with permission from Ref. [19]. Copyright 2016, American Chemical Society.

3D printing, also referred to as additive manufacturing, is an umbrella term that covers several digital model-based layer-by-layer deposition techniques. Among them, fused deposition modelling (FDM), direct ink writing (DIW), inkjet printing and stereolithography (SLA) have been successfully applied for fabrication of complex 3D structures of ECHs [19]. An overview of the basic operating principles of these techniques is illustrated in Figure 4, and their respective advantages and limitations are discussed in the following sub-sections.

### 3.1. Fused Deposition Modelling (FDM)

FDM is an extrusion-based printing method, where a filament made of thermoplastics is heated to reach a semi-liquid state and extruded through a nozzle, followed by self-solidification upon cooling on the printed platform, to form a solid layer on top of another [19]. The advantages of this method include low cost and simplicity, whereas the main disadvantage is the relatively low printing resolution. Moreover, FDM is not suitable for bioprinting, thereby making it the least favourable method for printing of ECHs.

### 3.2. Direct Ink Writing (DIW)

DIW is also an extrusion-based printing method, where a shear-thinning viscous liquid or paste is extruded (under pressure) through the nozzle, followed by solidification via evaporation of solvent, rapid cryogenisation, sol-gel transition, crosslinking or post-treatment on the printed platform, to form a solid layer on top of another [19]. DIW is more suitable and widely applied method for printing of soft hydrogels, especially for biomedical applications, due to the broad choices of materials, provided that the deposited ink can be rapidly solidified. A variety of materials can be used for fabrication of ECHs using this method, ranging from pristine polymers [20] to composite materials [21]. Moreover, DIW is the only printing technology that has been successfully applied for 3D bioprinting of ECHs [22]. The rheological properties of the inks play a crucial role in the DIW process. A 3D printable ink usually requires shear-thinning characteristics, wherein the ink pastes exhibit low elastic shear modulus under high shear stress to flow through the nozzle and high static elastic modulus to maintain its shape after deposition for printing of multiple layers [23]. DIW is the most popular technique for 3D printing of ECHs; however, its main drawback is also the low printing resolution.

### 3.3. Inkjet Printing

In inkjet printing, the ink droplets are propelled through a nozzle by a thermal/piezoelectric actuator and selectively deposited on demand to the desired location of a substrate. Precise deposition of small droplets helps to print high-resolution structures, followed by solidification of the printed droplets by chemical or thermal process such as curing or sintering [19]. Inkjet 3D printed ECHs can be fabricated by sequential deposition or spray-coating (on a substrate covered by a patterned mask) of two distinct solutions consisting of (i) monomer precursor and (ii) oxidising agent in a layer-by-layer fashion, with masks placed on previous layer [24]. However, inkjet printing of ECHs is challenging due to the strict requirements for ink formulation regarding its viscosity, surface tension and the evaporation rate.

### 3.4. Stereolithography (SLA)

In SLA, photocurable polymer solution (that is contained in a reservoir) is converted into photopolymerized solid in a layer-by-layer fashion using light energy (e.g., UV or laser beam) [19]. Currently, SLA is used to achieve the best resolution printing of ECHs, and it can work with a wide variety of materials [25]. However, its drawbacks include conducting polymer’s intense absorption (due to the presence of long chains of conjugated π bonds) in the UV and visible region, and their complex redox chemistries, which may be incompatible with the reactive species formed during some photoinitiator decomposition [19].

## 4. State-of-the-Art 3D Printed ECHs: Fabrication, Properties and Biomedical Applications

### 4.1. Conducting Polymer-Based Gel

The main challenge for the development of ECHs for biomedical applications is to achieve high conductivity, while not compromising its physicochemical properties and biocompatibility. For these reasons, ECHs based on biocompatible conductive polymers, such as PEDOT:PSS, PANI and PPy, which exhibit a perfect blend of biocompatibility, high conductivity and required mechanical properties, have made significant progress in tissue engineering [7]. Table 1 provides the summary of 3D printed electrically conductive polymer-based hydrogels and scaffolds that can be potentially applied in the field of biomedical engineering.

**Figure 5:**
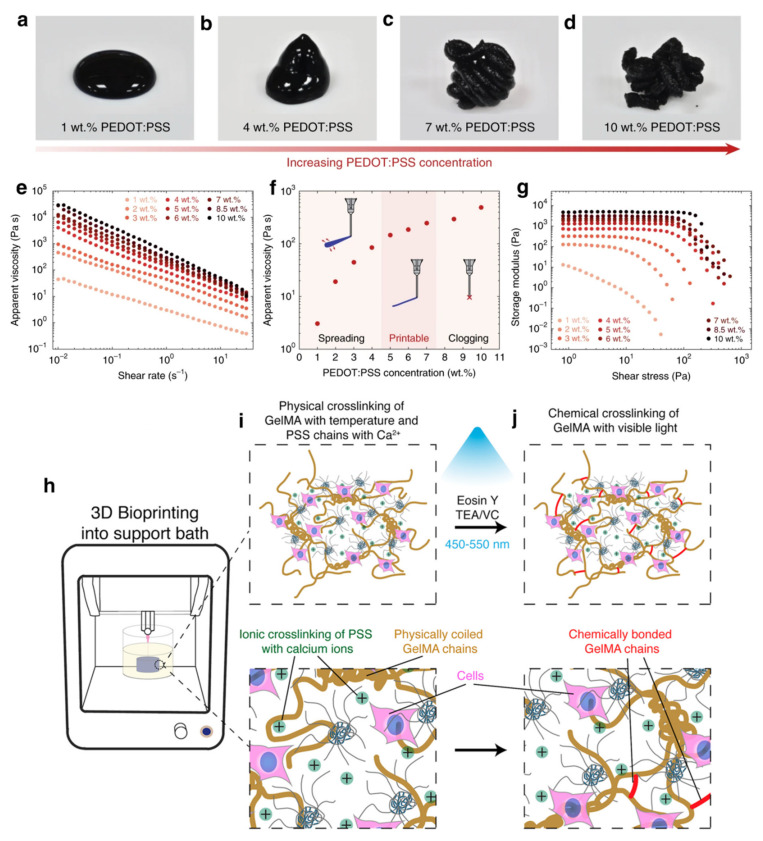
(**a**–**d**) Images of redispersed suspensions of PEDOT:PSS nanofibrils at various concentrations. (**e**) Apparent viscosity of PEDOT:PSS nanofibril suspensions as a function of (**e**) shear rate and (**f**) concentration. (**g**) Shear storage modulus of PEDOT:PSS nanofibril suspensions as a function of shear stress. Adapted with permission from Ref. [20], Copyright 2020, Springer Nature. (**h**) Schematic of 3D bioprinting of cell-laden GelMA/PEDOT:PSS bioink into a coagulation bath containing aqueous calcium chloride at 4 °C. (**i**) Physical and ionic crosslinking in 3D bioprinted GelMA/PEDOT:PSS structures. (**j**) Photo-cross-linking of GelMA chains in 3D bioprinted GelMA/PEDOT:PSS structures. Adapted with permission from Ref. [22]. Copyright 2019, American Chemical Society.

PEDOT:PSS is one of the most promising conducting polymers used in ECH bioelectronics owing to its high electrical conductivity and water dispersibility [26]. While DIW 3D printing of pure PEDOT:PSS hydrogels has remained a challenge in the last decade, rapid cryogenisation of aqueous PEDOT:PSS dispersions followed by lyophilization and controlled redispersion has been recently reported to give favourable rheological properties (Figure 5a–g) for a 3D printable PEDOT:PSS ink with high biocompatibility [20], which has also been patented for industrial translation [27]. This is an effective approach to scalable and rapid fabrication of soft neural probe capable of in vivo single-unit recording. In order to communicate with the nervous system, an electrical coupling of the electrode with the neural tissue is required. Ionic and/or electronic charge transfer across the electrode-tissue interface captures or stimulates neural activity. Thus, soft electrodes based on conductive hydrogels (ECHs) are desirable, which can improve the viability of neural cells and long-term performance of bionic devices. Moreover, the ECHs can encapsulate cells which can integrate with the surrounding tissue, forming an intimate connection and preventing scar tissue growth. Development of scar tissue also leads to higher amounts of charge being required to activate the target tissue over time, reducing implant efficacy. Recently, 3D printed PEDOT:PSS structures have been converted into a soft (Young’s modulus below 1.1 MPa) and highly conductive (conductivity up to 28 S cm^−1^) hydrogels through subsequent swelling in a wet environment, which was also tested to be biocompatible with mouse dorsal hippocampus [20]. In order to harness the specific property advantages of a wide range of polymeric biomaterials available, such as poly(ethylene glycol) diacrylate (PEGDA), gelatin methacrylate (GelMA), methyl cellulose (MC), kappa-carrageenan (kCA), poly(2-hydroxyethyl methacrylate) (PHEMA), poly(ethylene glycol methacrylate) (PEGMA) and their copolymer p(HEMA-co-EGMA) [28], several PEDOT:PSS-based hybrid ECHs have been investigated for 3D printability. Lately, PEGDA/PEDOT:PSS hybrid hydrogels that can be photocrosslinked while maintaining their high electrical conductivity weere fabricated using SLA 3D printing. The printed structures exhibited tuneable electrical resistance (0.7 to 2.8 kΩ/sq) based on the PEDOT:PSS content and were capable of transferring electrical stimulations towards encapsulated dorsal root ganglion neuronal cells for enhanced neuronal differentiation, suggesting possible applications in interfacial bioelectronics with biological stimulation to regulate and induce cellular behaviour [29]. SLA has also been used for 3D printing p(HEMA-co-EGMA)/PEDOT:PSS hybrid ECHs exhibiting compressive modulus of 80 kPa and good biocompatibility with neural progenitor PC-12 cells, which has potential applications in neural tissue engineering [30]. Conversely, DIW 3D bioprintable GelMA/PEDOT:PSS hybrid ink has been developed for designing complex cell-laden electroactive structures, which can be ionically crosslinked in calcium chloride (CaCl_2_) bath and photocrosslinked to form stable living structures (Figure 5h–j). The 3D bioprinted (with mouse myoblast C2C12 cell) and crosslinked hybrid hydrogels exhibited tuneable mechanical stiffness (tensile modulus of 40.9 to 141.7 kPa) and electrical resistance (261.0 to 281.2 kΩ), with good water swelling ratio (600 to 1100%) and biocompatibility both in vitro (>95% viability) and in vivo [22]. In applications requiring cell transplantation for electroactive tissue regeneration, the high conductivity of the printed structures can be useful for increasing the bioelectronic function of the tissue during the regeneration process. However, the photocrosslinking process also suffers from endogenous oxidative damage of DNA and the decreased cell viability by prolonged light exposure [31]. To address this issue, a highly thixotropic and conductive bioink was prepared by first blending kCA with PEDOT:PSS, followed by dispersion of MC in the kCA/PEDOT:PSS blend for fabrication of DIW 3D printable physiological-scale structures of MC/kCA/PEDOT:PSS hybrid ECHs with high resolution and high shape fidelity without the need for light curing. As a result, the 3D bioprinted (human embryonic kidney cells) and ionically crosslinked (in aqueous potassium chloride bath) hybrid ECHs exhibited compressive modulus in the range of 8.0–28.5 kPa, excellent water swelling ratio (3000 to 5800%) and biocompatibility (>96% viability) [31]. The above-developed PEDOT:PSS-based bioinks and 3D bioprinted ECHs have tremendous potential for a wide range of tissue engineering applications. Recently, a novel strategy based on melt processing that enables FDM 3D printing of Nafion template followed by polymerization of EDOT monomer within the printed template has been developed. The resulting nontoxic structures, where nafion has been reported to have good biocompatibility in mice models [32], are highly flexible, exhibiting conductivity of ~3 S cm^−1^ upon stretching to 100% elongation, which has opened up a new possibility for the design of all-polymer ECH bulk structures for the development of wearable electronics, e-textiles and biosensors [33]. 

PPy is a conjugate polymer with high environmental stability, tunable conductivity and biocompatibility, which has potentially application in tissue engineering; however, is rigid, insoluble and non-biodegradable [7]. Therefore, hybridization of PPy with other biocompatible and biodegradable polymers, such as poly(acrylic acid) (PAA), chitosan (Chi), Poly-l-lactide (PLLA) and alginate (Alg) [28], can tune its properties and expand its use as promising ECHs for bioelectronics. Recently, DIW 3D printing of double network PAA/PPy-Chi hybrid hydrogel structures, with acrylic acid polymerisation for mechanical integrity and ionic crosslinking between PAA and PPy-grafted chitosan (PPy-Chi) for self-healing has been reported. The 3D printed hybrid ECHs exhibited electrical conductivity in the range of 13.0 to 30.0 S cm^−1^ with 1500% stretchability [34]. Such systems have potential for strain and pressure-sensing wearable device applications. Chemical oxidative polymerization of PPy in preformed PEGDA structures by SLA 3D printing shows the fabricated hybrid ECH structures to exhibit electrical resistance in the range of 0.013 to 3.5 MΩ.cm based on the reaction conditions. This ECH shows promise for bioelectronic applications [35]. Conversely, PLLA/PPy hybrid hydrogels with tuneable conductivity and compression strength in the range of 170–750 mS/cm and 18–32 MPa, respectively, have been fabricated using DIW 3D printing, where preformed PPy nanoparticles were dispersed in the PLLA ink and printed on cold substrate (at −7 °C), followed by freeze-drying for structural integrity. The 3D printed ECHs showed excellent mouse L929 fibroblast cell viability and proliferation, which can be applied for a wide range of tissue engineering applications [36]. One of the key issues in such ECH device fabrication is the rapid crosslinking chemistry. The well-studied Ca^2+^ ion-based crosslinking of Alg has been employed to fabricate Alg/PPy hybrid ECHs by DIW 3D printing of Alg/PPy blends into an alcohol coagulation bath containing CaCl_2_. The 3D printed free-standing hydrogels exhibited excellent biocompatibility (with neuronal PC12 cells) and tuneable conductivity in the range of 4.1–6.3 mS cm^−1^, providing a potential route for spatially controlled cellular adhesion and growth for neural tissue engineering [37].

PANI is a robust conducting polymer from the semi-flexible rod polymer family, with ease of synthesis and biocompatible properties that has been potentially applied for tissue engineering [7]. Fabrication of 3D PANI hydrogels by inkjet printing of phytic acid and aniline solutions in layers on flexible substrates have been previously reported, which exhibited excellent electronic conductivity (~0.1 S cm^−1^) and superior glucose sensitivity [24]. However, the use of PANI in the development of free-standing 3D printable ECSs for tissue engineering is restricted due to its poor processability (low solubility in common solvents) and non-biodegradability [7]. To overcome these limitations, PANI can be hybridized with other biocompatible polymers, such as gelatin methacrylate (GelMA), polysulfone (PSU) and polycaprolactone (PCL) [28,38], paving the way for potential applications in bioelectronic interfaces and implantable devices. 3D GelMA/PANI hybrid ECHs have been fabricated by interfacial polymerization of aniline monomers into SLA-printed GelMA matrix. The hybrid hydrogels exhibited electrical resistance and compressive modulus around 165.6 Ω and 14.5 kPa, respectively, and good biocompatibility with murine mesenchymal progenitor 10T1/2s cells, which can be potentially applied for bioelectronic interfaces [25]. Such systems can also be potentially applied for DIW-based 3D printing of free-standing GelMA/PANI ECHs. In a separate study, PSU/PANI hybrid scaffolds were 3D printed (using DIW method) from PSU ink containing PANI powder dispersed. The 3D printed structures exhibited high electrical resistance with undoped PANI and exhibited semiconductor properties using doped PANI [39]. Such systems can be used to fabricate implantable biodevices with tuneable electrical properties. Conversely, PCL/PANI hybrid scaffolds were 3D printed (using FDM method) from PCL melt containing PANI powder dispersed. The 3D printed hybrid scaffolds exhibited compressive modulus of 6.45 ± 0.16 MPa, electrical conductivity of 2.46 ± 0.65 × 10^−4^ S cm^−1^, and outstanding human adipose-derived stem cell viability, which has potential for bone tissue engineering applications and implantable biodevices [40].

**Table 1:** Summary of 3D printed electrically conductive polymer-based hydrogels and scaffolds for biomedical applications.

Hydrogel Composition	Bioink	Printing Methods and Parameters	Crosslinking/Post-Treatment	Electrical Properties	Mechanical Properties	Biocompatibility	Biomedical Applications	Reference
PEDOT:PSS	No	DIW: Nozzle diameter—30, 50, 100 and 200 µm.	Air-drying, annealing	Conductivity—28.0 S cm^−1^ (wet), and 155.0 S cm^−1^ (dry).	Compressive modulus—1.1 MPa (wet), and 1.5 GPa (dry).	Mouse dorsal hippocampus	Neural tissue engineering	[20]
PEGDA/PEDOT:PSS	No	SLA: Laser spot diameter—200 µm; print speed—8 mm/s; UV wavelength—355 nm.	Photocrosslinking	Resistance—0.7 to 2.8 kΩ/sq.	Compression stiffness—26.3 to 35.4 MPa.	Dorsal root ganglion neuronal cell differentiation under electrical stimulation	Neural tissue engineering	[29]
p(HEMA-co-EGMA)/PEDOT:PSS	No	SLA; EB: Nozzle diameter—200 µm; print speed—2.5 to 8.0 mm/s	UV curing	Resistance—100 to 125 kΩ.	Compressive modulus—82 kPa.	Neural progenitor cells	Neural tissue engineering	[30]
GelMA/PEDOT:PSS	Yes	DIW: Nozzle diameter—160 µm; print speed—5 to 10 mm/s; pressure—70 to 90 kPa.	Printing in CaCl_2_ support bath, photocrosslinking, isotherm	Resistance—261.0 to 281.2 kΩ.	Tensile modulus—40.9 to 141.7 kPa.	Mouse myoblast cells, subcutaneous implant in rats	Tissue engineering	[22]
MC/kCA/PEDOT:PSS	Yes	DIW: Nozzle diameter—210 µm; print speed—1, 2, 4, 6 and 8 mm/s; pressure—10, 15 and 20 psi.	Immersion in 5 wt% KCl solution	Conductivity—1.2 to 2.9 mS cm^−1^.	Compressive modulus—8.0 to 28.5 kPa.	Human embryonic kidney cells	Tissue engineering	[31]
Nafion/PEDOT	No	FDM: Substrate printing	Interfacial polymerization of EDOT monomers into post-printed Nafion matrix	Conductivity—1 to 5 S cm^−1^ (dry)	Tensile modulus—620 MPa (dry).	-	Wearable sensors	[33]
PAA/PPy-Chi	No	DIW: Nozzle diameter—300 µm; print speed—2 mm/s; pressure—40 psi.	Washing with 5 wt% ammonium persulfate solution	Conductivity—13.0 to 30.0 S cm^−1^.	Compressive modulus—0.6 to 0.8 MPa; stretchability—1500%.	-	Wearable sensors	[34]
PEGDA/PPy	No	SLA: Substrate printing	Interfacial polymerization of pyrrole monomers into post-printed PEGDA matrix	Resistance—0.013 to 3.5 MΩ.cm.	Compressive modulus—0.6 to 1.4 MPa.	-	Bioelectronics	[35]
PLLA/PPy	No	DIW: Nozzle diameter—260 µm; print speed—140 mm/min; pressure—5 kPa.	Printing with receiving condenser at -7 °C, freeze-drying	Conductivity—170 to 750 mS cm^−1^.	Compressive strength—18 to 32 MPa.	Mouse fibroblast cells	Tissue engineering	[36]
Alg/PPy	No	DIW: Nozzle diameter—100 µm; print speed—140 mm/min; pressure—5 kPa.	Printing in 15% ethanol coagulation bath with 5% CaCl_2_	Conductivity—4.1 to 6.3 mS cm^−1^.	-	Neuronal cells	Neural tissue engineering	[37]
PANI	No	Inkjet: Microdot arrays—18 to 21.5 µm diameter; Nozzle diameter—9 to 40 µm.	In situ polymerization of aniline monomer	Conductivity—0.1 S cm^−1^.	-	-	Bioelectronics	[24]
GelMA/PANI	No	SLA: Substrate printing	Interfacial polymerization of aniline monomers into post-printed acidic GelMA matrix	Resistance—165.6 Ω.	Compressive modulus—13.7 to 15.2 kPa.	Murine mesenchymal progenitor cells	Bioelectronic interfaces	[25]
PSU/PANI	No	DIW: Nozzle diameter—600 µm; print speed—6.8 mm/s; pressure—93.6 psi.	-	Resistance—4.8 Ω.m (dry).	-	-	Implantable biodevices	[39]
PCL/PANI	No	FDM: Nozzle diameter—330 µm; print speed—20 mm/s; pressure—6 bar.	-	Conductivity—0.25 to 0.28 mS cm^−1^ (dry).	Compressive modulus—68.4 to 82.6 MPa (dry).	Human adipose-derived stem cells	Implantable biodevices	[40]

### 4.2. Conductive Filler-Based Gel

**Figure 6:**
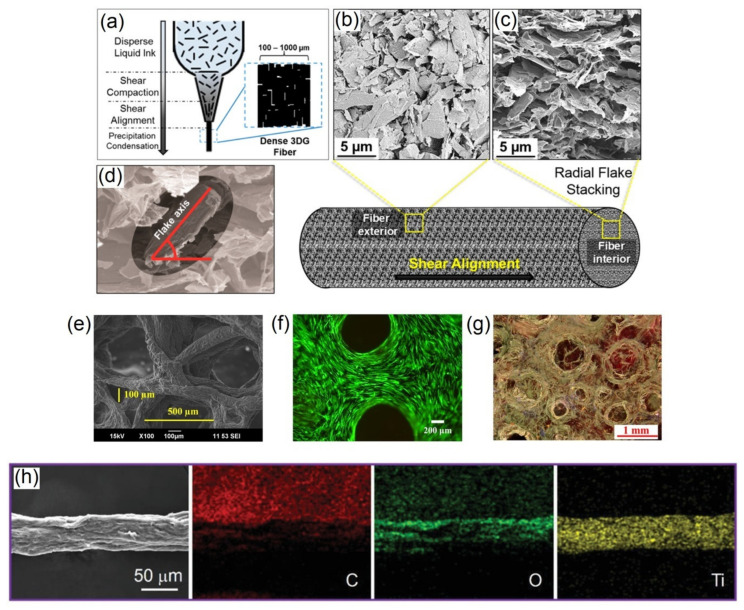
(**a**) Schematic of 3D printing PLG/pGn composite hydrogels showing graphene flake alignment with shear force. SEM images of the 3D printed fiber: (**b**) exterior (showing graphene flake alignment) and (**c**) cross-section (showing graphene flake stacking). (**d**) Graphene flake orientation in an end-on cross-sectional view of the 3D printed PLG/pGn composite fiber. Adapted with permission from Ref. [21]. Copyright 2015, American Chemical Society. (**e**) SEM image of 3D printed Alg/rGO composite hydrogel. (**f**) Fluorescence microscope image of human adipose stem cells (calcein/propidium iodide staining with live cells emitting green fluorescence) cultured for 7 days on 3D printed Alg/rGO composite hydrogel. (**g**) Light microscope image of mineral deposition (Alizarin Red S stained) by human adipose stem cells cultured for 7 days on 3D Alg/rGO composite hydrogel. Adapted with permission from Ref. [52]. Copyright 2020, Frontiers. (**h**) SEM images with elemental (C, O and Ti) mapping of TOCNFs/Ti_3_C_2_-Mxene composite fibres. Adapted with permission from Ref. [53]. Copyright 2019, Wiley.

The emergence of conductive filler materials with a high level of anisotropic activity and chemical characteristics can provide new approaches for the design and development of versatile and high-performance bioelectronic materials with new functionalities [8]. In particular, graphene, MXenes and liquid metals have become the three most effective conductive filler materials applied in advanced ECHs [9,10,11]. In the last decade, graphene, a single-layer carbon allotrope with a two-dimensional honeycomb lattice atomic arrangement has become one of the most effective conductive fillers for advanced ECHs due to its outstanding mechanical properties, tuneable electrical conductivity and biocompatibility [41]. To fabricate graphene-based composite ECHs for biomedical application, the carbon allotropes—graphene oxide (GO), reduced graphene oxide (rGO) and pristine graphene or graphene nanoplatelets (GNPs)—are commonly dispersed in biocompatible and biodegradable polymers, such as chitosan (Chi), poly(lactide-co-glycolide) (PLG), poly(ethylene glycol) (PEG), polyethylenimine (PEI), chitosan methacrylate (ChiMA), poly(lactic acid) (PLA), poly(vinyl butyral) (PVB), hydroxypropyl cellulose (HPC), poly(acrylic acid) (PAA) and gelatin (Gel) [28], which provide structural integrity and water sorption property. Table 2 provides a summary of 3D printed conductive graphene, MXene and liquid metal-based ECHs and scaffolds that can be potentially applied in the field of biomedical engineering. In one of the first attempts to 3D printed graphene-based composite ECHs, rGO was DIW 3D printed with chitosan in an isopropyl alcohol precipitating bath where the fabricated ECHs exhibited tuneable conductivity and tensile strength (wet) in the range of 0.01–15.00 µS m^−1^ and 272–372 kPa based on rGO/polymer weight ratio. Moreover, the ECHs exhibited water swellability in the range of 110–260% and excellent biocompatibility with L929 mouse fibroblast cells [42]. Such systems can be potentially applied as conducting substrates for the growth of electro-responsive cells in tissue engineering. In a separate study, when GNPs (60 vol%) were dispersed in PLG matrix and DIW 3D printed, the printing shear force resulted in GNPs to alignment/orient to the printing direction, as shown in Figure 6a–d. The 3D printed composite ECH structures exhibited high electrical conductivity (875 S cm^−1^) and good biocompatibility with human mesenchymal stem cells [21]. The composition of PLG/GNPs was later expanded to include hydroxyapatite (Hap) to form PLG/Hap/GNPs composite ECHs; however, this reduced the conductivity (127 S cm^−1^) of the DIW 3D printed composite ECHs [43]. In a separate study, ChiMA has been used as a polymer matrix for dispersing rGO and DIW 3D printing ChiMA/rGO composite ECHs in a precipitation bath of isopropanol, followed by UV crosslinking. The 3D printed composite ECHs exhibited tuneable conductivity and water swelling in the range of 20–250 µS cm^−1^ and 160–205%, with excellent L929 mouse fibroblast cell biocompatibility [44]. The above 3D printed systems have potential for a wide range of tissue engineering applications. Moreover, a polymer blend solution of PEG/PEI has also been used to disperse GNPs and DIW 3D printed into PEG/PEI/GNPs composite structures. The as-fabricated ECHs exhibited a conductivity of 5.6 S cm^−1^, which increased to 533.5 S cm^−1^ with spark plasma sintering and burning out (475 °C) in air [45]. Lately, SLA method has also been reported for fabrication of rGO filler-based composite ECHs, where PEGDA/GO composite formulation was 3D printed and photocrosslinked, followed by a thermal post-treatment to obtain PEGDA/rGO composite ECHs. The PEGDA/rGO composite ECHs exhibited conductivity and compressive modulus in the range of 95.8–109.5 nS cm^−1^ and 6.8–8.7 MPa, respectively [46]. The above 3D printed composite ECHs have potential for bioelectronics applications.

FDM has also been successfully applied to 3D print GNPs and rGO filler-based electrically conducting scaffolds. For instance, PLA/GNPs composite scaffolds were 3D printed at 260 °C on a 60 °C substrate, which exhibited an electrical resistance of 102 Ω.cm [47], whereas PCL/rGO composite scaffolds were-3D printed at 220 °C, which exhibited a conductivity of 0.68 µS m^−1^ [48]. Furthermore, the FDM 3D printed PCL/rGO composite scaffolds showed a strong antibacterial effect via completely eradicating *S. aureus* under electrical stimulation, whereas showed increased viability of human bone marrow-derived mesenchymal stem cells [48]. The above 3D printed composites can be potentially applied in bioelectronics and hard tissue engineering. Recently, surfactant-stabilized GNPs were dispersed in PVB solution and DIW 3D printed to obtain PVB/GNPs composite scaffolds and exhibited conductivity and compressive modulus in the range of 130–230 S m^−1^ and 0.57–4.37 MPa, respectively [49]. Conversely, when a mixture of Fe_3_O_4_ functionalized GNPs and HPC was DIW 3D printed and thermally annealed at different temperatures, the obtained HPC/Fe_3_O_4_-GNPs composites exhibited conductivity in the range of 85–580 S m^−1^. In addition, the 3D printed composite also exhibited a saturation magnetization of 15.8 emu/g [50]. The above 3D printed composite systems have potential for bioelectronics and biosensor applications. Recently, PAA/GO composite systems with calcium ions to bridge adjacent GO and PAA/GO through COO^−^ groups have been DIW 3D printed and hydroiodic acid vapour-treated (to reduce GO) to obtain PAA/rGO composite ECH structures, which exhibited electrical resistance in the range of 230–855 kΩ [51]. The 3D printed PAA/rGO composites have potential in wearable sensors, actuators and bioelectronics applications. In a separate study, Alg/Gel blends were DIW 3D printed in CaCl_2_ support bath, followed by dissolution of Gel in water at 80 °C and GO coating/incorporation on resulting porous construct. The GO was then reduced using ascorbic acid to obtain Alg/rGO ECHs with electrical resistance of 1.5 kΩ/sq and compressive modulus of 195 kPa. Moreover, the fabricated composite ECHs showed excellent human adipose stem cell viability and mineral deposition (as shown in Figure 6e–g), which has potential in tissue engineering as support for osteogenic induction [52].

MXenes are layers of transition metal carbides, carbonitrides or nitrides a few atoms thick and have attracted attention in the field of biomedical engineering due to their aqueous dispersion processability, tuneable electrical conductivity and biocompatibility [8]. Preparation of additive-free aqueous and organic inks of Ti_3_C_2_T_x_ has been recently reported for extrusion and inkjet printing, respectively, of micro-supercapacitors on flexible substrates with resistance values ranging from a few Ω to several MΩ [54]. However, the fabricated structures are brittle and require blending with biopolymers, such as Tempo oxidised cellulose nanofibers (TOCNF) and hyaluronic acid (HA) [28], to tune their mechanical strength, biocompatibility and free-standing scaffold structure processability. TOCNF/Ti_3_C_2_-MXene composite ECH fibres and textiles DIW 3D printed in alcohol coagulation bath have shown remarkable rapid photothermal, electrothermal and electromechanical responsiveness, with conductivity and tensile modulus tuneable in the range of 4.8–211.0 S m^−1^ and 4.7-9.3 GPa, respectively. SEM images with elemental mapping showing uniform distribution of Ti_3_C_2_-Mxene in fabricated TOCNFs/Ti_3_C_2_-Mxene composite fibers are shown in Figure 6h [53]. These smart fibres and textiles are promising for the development of next-generation healthcare electronics, including wearable heating and sensing systems. Conversely, a bioink containing a mixture of HA, Alg and Ti_3_C_2_-MXene nanosheets with human embryonic kidney 293 cells were DIW 3D bioprinted in aqueous CaCl_2_ bath. The highly thixotropic behavior of HA/Alg/Ti_3_C_2_-MXene mixture offered excellent printability of the bioink and the fabricated composite ECHs exhibited excellent viability (>95%), and electrical conductivity and compressive modulus in the range of 1.2–7.2 mS cm^−1^ and 2.8–5.5 kPa, respectively [55]. Such systems indicate the exciting potential of 3D printable MXene-based bioinks for neural tissue engineering applications.

Liquid metals (LMs) are family of functional metal and metal alloys, which are liquid at or near room temperature offering fluidic flexibility in shape and size in addition to standard metallic properties. In recent years, gallium (Ga)—known to have low toxicity and near-zero vapour pressure at room temperature, and Ga-based eutectic alloy LMs (alloyed with indium (In) and tin (Sn)) exhibiting therapeutic properties, such as anticancer and antimicrobial, have emerged as a promising material in the field of biomedical engineering [10]. Because of their fluidic nature, fabrication of 3D structures of LMs requires mixing them with biopolymers and polyphenols, such as alginate (Alg) and tannic acid (TA) [28], to support processability into free-standing structure and biocompatibility. The first attempt to 3D print LMs involved a conceptual method, namely suspension of 3D printing microdroplets of LM (eutectic alloy of Ga and In, EGaIn) into a supporting and self-healing carbopol hydrogel medium [56]. Recently, Alg/EGaIn-LM composite inks were developed as core-shell (EGaIn core, Alg shell) aqueous microgel dispersion, which was inkjet-printed and mechanically sintered to obtain patterns with conductivity of 0.4 MS m^−1^ [57]. In a separate study, TA/EGaIn-LM composite dispersions were used as inks in a ballpoint pen for writing conducting patterns using a DIW 3D printer. The fabricated complex patterns of the TA/EGaIn-LM composite exhibited conductivity in the range of 0.29-1.6 MS m^−1^ [58]. The above fabricated systems have great potential for flexible biosensor and bielectronics applications.

**Table 2:** Summary of 3D printed electrically conductive filler-based hydrogels and scaffolds for biomedical applications.

Hydrogel Composition	Bioink	Printing Methods and Parameters	Crosslinking/Post-Treatment	Electrical Properties	Mechanical Properties	Biocompatibility	Biomedical Applications	Reference
Chitosan/rGO	No	DIW: Nozzle diameter—200 µm; print speed—150 mm/min.	Printing in isopropyl alcohol precipitating bath	Conductivity—0.015 to 15 µS m^−1^ (dry).	Tensile strength—272 to 372 kPa.	Mouse fibroblast cells	Tissue engineering	[42]
ChiMA/rGO	No	DIW: Nozzle diameter—200 µm; print speed—150 mm/min.	Printing in isopropyl alcohol precipitating bath	Conductivity—20 to 250 µS m^−1^ (dry).	-	Mouse fibroblast cells	Tissue engineering	[44]
PEGDA/rGO	No	SLA: 50 µm projector resolution, print time—1.5 to 2 s/layer.	UV curing, thermal reduction of GO	Conductivity—95.8 to 109.5 n S cm^−1^.	Compressive modulus—6.8 to 8.7 MPa (dry).	-	Bioelectronics	[46]
PCL/rGO	No	FDM: Nozzle diameter—0.9 mm; print speed—0.3 mm/s; temperature 220 °C; pressure—6 bar.	Air-drying	Conductivity—0.68 µS m^−1^ (dry).		Human bone marrow-derived mesenchymal stem cells	Tissue engineering, anti-bacterial	[48]
PAA/rGO	No	DIW: Nozzle diameter—600 µm.	Humidity curing, hydroiodic acid vapor induced reduction of GO	Resistance—230 to 855 kΩ.	-	-	Wearable	[51]
Alg/rGO	No	DIW: Substrate printing	Printing Alg/Gel, immersion in CaCl_2_ bath, Gel dissolution by thermal treatment, GO coating/incorporation, ascorbic acid induced reduction of GO	Resistance—1.5 kΩ/sq	Compressive modulus—195 kPa.	Human adipose stem cells	Tissue engineering	[52]
PLG/GNPs	No	DIW: Nozzle diameter—410 µm; print speed—10 to 45 mm/s; pressure—0.5 to 5.0 bar.	-	Conductivity—875 S m^−1^ (dry).	Compressive modulus—3.0 MPa (dry).	Human mesenchymal stem cells, subcutaneous implant in mouse	Nerve guide conduits	[21]
PLG/Hap/GNPs	No	DIW: Nozzle diameter: 100, 200 400 and 1000 µm, print speed: 10 to 75 mm/s; pressure—0.5 to 5.0 bar.	-	Conductivity—127 S cm^−1^ (dry).	Compressive modulus—3.0 MPa (dry).	Human mesenchymal stem cells	Tissue engineering	[43]
PEG/PEI/GNPs	No	DIW: Nozzle diameter—400 µm.	Spark plasma sintering, burning-out	Conductivity—5.6 to 533.5 S cm^−1^ (dry).	Compressive modulus—0.35 to 0.58 MPa (dry).	-	Bioelectronics	[45]
PLA/GNPs	No	FDM: Nozzle diameter—0.4 mm; temperature 210 °C.	Air-drying	Resistance –102 Ω.cm (dry).	Tensile modulus—2.4 GPa (dry).	-	Bioelectronics	[47]
PVB/GNPs	No	DIW: Nozzle diameter—300, 400 and 500 µm; print speed—5 to 15 mm/s; pressure—0.1 to 0.6 MPa.	Air-drying	Conductivity—130 to 230 S m^−1^ (dry).	Compressive modulus—0.57 to 4.37 MPa (dry).	-	Bioelectronics	[49]
HPC/Fe_3_O_4_-GNPs	No	DIW: Nozzle diameter—200 µm; print speed—1 mm/s; pressure—140 psi.	Annealing at different temperatures	Conductivity—85 to 580 S m^−1^ (dry).	-	-	Biosensors	[50]
TOCNF/Ti_3_C_2_-MXene	No	DIW: Nozzle diameter—600 µm; print speed—4.2 mm/s.	Immersion in ethanol coagulation bath	Conductivity—4.8 to 211.0 S m^−1^ (dry).	Tensile modulus—4.7 to 9.3 GPa (dry).	-	Wearable sensors	[53]
HA/Alg/Ti_3_C_2_-MXene	Yes	DIW: Nozzle diameter—210 µm; print speed—6 mm/s; pressure—5 and 15 psi.	Immersion in CaCl_2_ bath	Conductivity—1.2 to 7.2 mS cm^−1^ (ink).	Compressive modulus—2.8 to 5.5 kPa.	Human embryonic kidney cells	Tissue engineering	[55]
Alg/EGaIn-LM	No	Inkjet	Mechanical sintering	Conductivity—0.4 MS m^−1^ (dry).	-	-	Bioelectronics	[57]
TA/EGaIn-LM	No	DIW: Writing speed—2.5 mm/s.	Air-drying	Conductivity—0.29 to 1.6 MS m^−1^ (dry).	-	-	Bioelectronics	[58]

### 4.3. Hybrid Methods for Fabrication of Multiscale 3D Printed ECH Structures

Fabrication of hydrogels and scaffolds with a controlled geometric footprint (nano- to macroscale hierarchical structures) isa key goal in tissue engineering. Such complex structures can be achieved by combining 3D printing with other fabrication methods, such as electrospinning [59] and electrochemical deposition [60]. Electrospinning is a versatile electrohydrodynamic (EHD) method for fabrication of 3D nanofibrous scaffolds. The process involves application of a high electrostatic voltage to dropping polymer solution, which forms a conical jet (Taylor cone) by charge repulsion at the end of capillary and ejects fibres overcoming surface tension. The ejected fibres are collected on an electrically grounded substrate with solvent evaporated in air during jetting [61]. Fabrication of hydrogels and scaffolds combining 3D printing and electrospinning can overcome the limitations of their respective structures, such as low print resolution and nanopores, respectively, and mechanical properties. Four different combinations, such as (i) 3D printing on electrospun membrane [59], (ii) 3D printed scaffold coated with electrospun fibers [62], (iii) infusion and crosslinking of dispersed electrospun fibers into 3D printed scaffold [63] and (iv) layer by layer gluing and/or deposition of alternating 3D printed grid and electrospun membrane [64,65] have been reported. Recently, fabrication of first hybrid hydrogel structures with alternative layers of 3D printed PEO/PEDOT:PSS features and electrospun PLLA nanofibrous mesh was reported [66]. Subsequently, a single-layer coating of electrospun PEDOT:PSS and PANI on 3D printed PLA and PU substrates, respectively was also reported, where PANI nanofibers showed good adhesion on 3D printed PU structures, which can be potentially applied for fabrication of biodevices with conductive coating and tuneable water contact angle [67]. 

EHD cojetting is a relatively new method for 3D printing regularly tessellated nanostructures. The method involves 3D printing conducting polymer solutions on a conducting substrate with electrostatic potential applied between conducting capillary and substrate [68]. It can be potentially applied for 3D printing of flexible bioelectronic devices, such as 100 layers of PEO/PEDOT:PSS ECHs with tuneable resistance in the range of 1–16 kΩ cm^−1^ [66]. In a separate study, 3D printed PCL/PPy scaffolds were fabricated using this method, which exhibited conductivity and tensile modulus in the range of 0.28–1.15 mS cm^−1^ and 35–51 MPa, respectively, and supported proliferation and maturation of human embryonic stem cells-derived neural crest stem cells to peripheral neurons [69]. Such systems have the potential for porous nerve guide conduit applications. Conversely, 3D printing PLCL microfiber structures by EHD cojetting, followed by GO coating and its subsequent reduction to rGO by ascorbic acid, has been successfully demonstrated as scaffolds (with conductivity ~0.95 S cm^−1^) for neural regeneration. The fabricated scaffolds exhibited good biocompatibility with rat pheochromo-cytoma PC-12 cells [70]. On the other hand, in electrochemical 3D printing method, polymeric structures are deposited (by redox reaction) on working electrode surface exposed to conducting monomer/ion solution (with counter and reference electrode inserted) in syringe [60]. So far, there has been only one report of fabrication of conducting polymer-based 3D scaffold using this method, where PANI was selectively 3D printed by electropolymerization from a solution containing aniline monomer and copper ions [71]. However, this method is limited by its slow printing speed. Both EHD cojetting and electrochemical 3D printing are at their infancy and expected to grow in the coming years.

## 5. Conclusions and Outlook

ECHs and 3D printing are at the forefront of research and development of future bioelectronics, implants and medical devices. The advancement of hydrogel bioelectronics would primarily benefit from rationally driven design concepts that comprise the basic mechanisms of tissue–electrode interactions to achieve patient-specific healthcare and precision biomedicine. While carefully designed ECHs have the potential to offer the electrochemical stability, mechanical durability and ongoing biological performance close to that of biological tissues, 3D printing offers a versatile platform for design and construction of customized implants and medical devices. ECHs, made by embedding various conducting components, such as conductive polymers, carbon materials and metal nanomaterials, into hydrophilic hydrogel matrix, are a part of “smart biomaterial platform”, which has promise for bridging the interface between biology and electronics. These conductive materials offer great advantage in hydrogel bioelectronics and tissue engineering with their tuneable physical, chemical and electrical properties and noncytotoxicity, which can be tailored to specific needs and applications such as ideal material for drug release and neural and muscular tissue engineering. Although more than 25 conductive polymers have been developed over the years [16], PEDOT, PANi and PPy are arguably the most studied polymers for bioelectronics due to their good biocompatibility and cellular response; however, they are hydrophobic in nature. Conversely, new-generation conductive materials, such as graphene, MXenes and liquid metals, are excellent and attractive fillers for ECHs, which offer a wide range of the flexible properties, including hydrophobicity to hydrophilicity, semi-conducting to conducting and liquid-like to solid-like. Most of the reported ECHs do not have multiple biological functions, such as charge transport, stretchability, degradation control, biosensing, tissue regeneration and self-healing, which need to be improved in the future. The availability of a wide range of 3D printing methods, including FDM, DIW, inkjet and SLA provides significant opportunity for use with different types of materials and chemical/physical reactions. Despite the impressive rise in 3D printing of ECHs in recent years (Table 1 and Table 2), several possibilities and needs have not yet been achieved or addressed. In particular, 4D printing of ECHs systems remains unexplored. Such developments are possible by formulating systems with near-infrared light sensitive rGO [72], pH-responsive poly(2-vinylpyridine) [73], temperature-responsive poly(N-isopropylacrylamide) [74], hydration/solvent-responsive PEG/PEGDA hybrids [75], electric field-responsive ionized PAA [76], etc., which could be potentially applied for flow regulating biodevices and soft robotic bioactuators [77]. In addition, new 3D printing-based hybrid methods, such as EDH cojetting and electrochemical 3D printing, need to be further explored for different materials and formulations. Moreover, applying external stimuli, such as magnetic and acoustics, during 3D printing can further assist the control of orientation, alignment, distribution and assembly of conducting fillers and polymers within the 3D printed structures. Further efforts need to be made to produce more bioink formulations with appropriate material combination and customizable mechanical, electrical, chemical and biological properties of 3D bioprinted constructs. In addition, 3D bioprinting technologies will continue to improve the resolution, speed and compatibility with biomaterials. Furthermore, 3D printed ECHs have primarily focused on their therapeutic effects mostly in vitro, whereas in vivo studies have been rarely conducted using mouse models. 3D bioprinted cell-laden ECHs organ models are still at their infancy, and their long-term stability, functionality and cytocompatibility require further study before they can be implemented in clinical therapies.

## Data Availability

Not applicable.

## Abbreviations

Alg	Alginate
Chi	Chitosan
ChiMA	Chitosan methacrylate
TOCNF	Tempo oxidised cellulose nanofibers
DIW	Direct ink writing
EHD	Electrohydrodynamic
FDM	Fused deposition modelling
GO	Graphene oxide
Gel	Gelatin
GelMA	Gelatin methacrylate
GNPs	Graphene nanoplatelets
HA	Hyaluronic acid
Hap	Hydroxyapatite
HPC	Hydroxypropyl cellulose
kCA	Kappa-carrageenan
MC	Methylcellulose
PAA	Poly(acrylic acid)
PCL	Polycaprolactone
PEDOT	Poly(3,4-ethylenedioxythiophene)
PEG	Poly(ethylene glycol)
PEGDA	Poly(ethylene glycol) diacrylate
PEI	Polyethylenimine
PEGMA	Poly(ethylene glycol methacrylate)
PEO	Poly(ethylene oxide)
PHEMA	Poly(2-hydroxyethyl methacrylate)
PLA	Poly(lactic acid)
PLCL	Poly(l-lactic acid-co-caprolactone)
PLG	Poly(lactide-co-glycolide)
PLLA	Poly(l-lactide)
PPy	Polypyrrole
PSS	Polystyrene sulfonate
PSU	Polysulfone
PU	Polyurethane
PVA	Poly(vinyl alcohol)
PVB	Poly(vinyl butyral)
rGO	Reduced graphene oxide
SLA	Stereolithography
TA	Tannic acid
